# Supplementary material for: DMRforPairs: identifying Differentially Methylated Regions between unique samples using array based methylation profiles
Source: BMC Bioinformatics. 2014 May 15;15:141. doi: 10.1186/1471-2105-15-141 (PMC4046028; doi:10.1186/1471-2105-15-141)
Supplement: Additional file 2 — DMRforPairs output for the comparison of A431-MCF7 and NA17018-NA17105. Please start from the HTML files in each folder. Available via the BMC Bioinformatics website. [file 1471-2105-15-141-S2.zip › 1394847754114233_MOESM2_ESM/A431_MCF7/figures/35473.pdf]

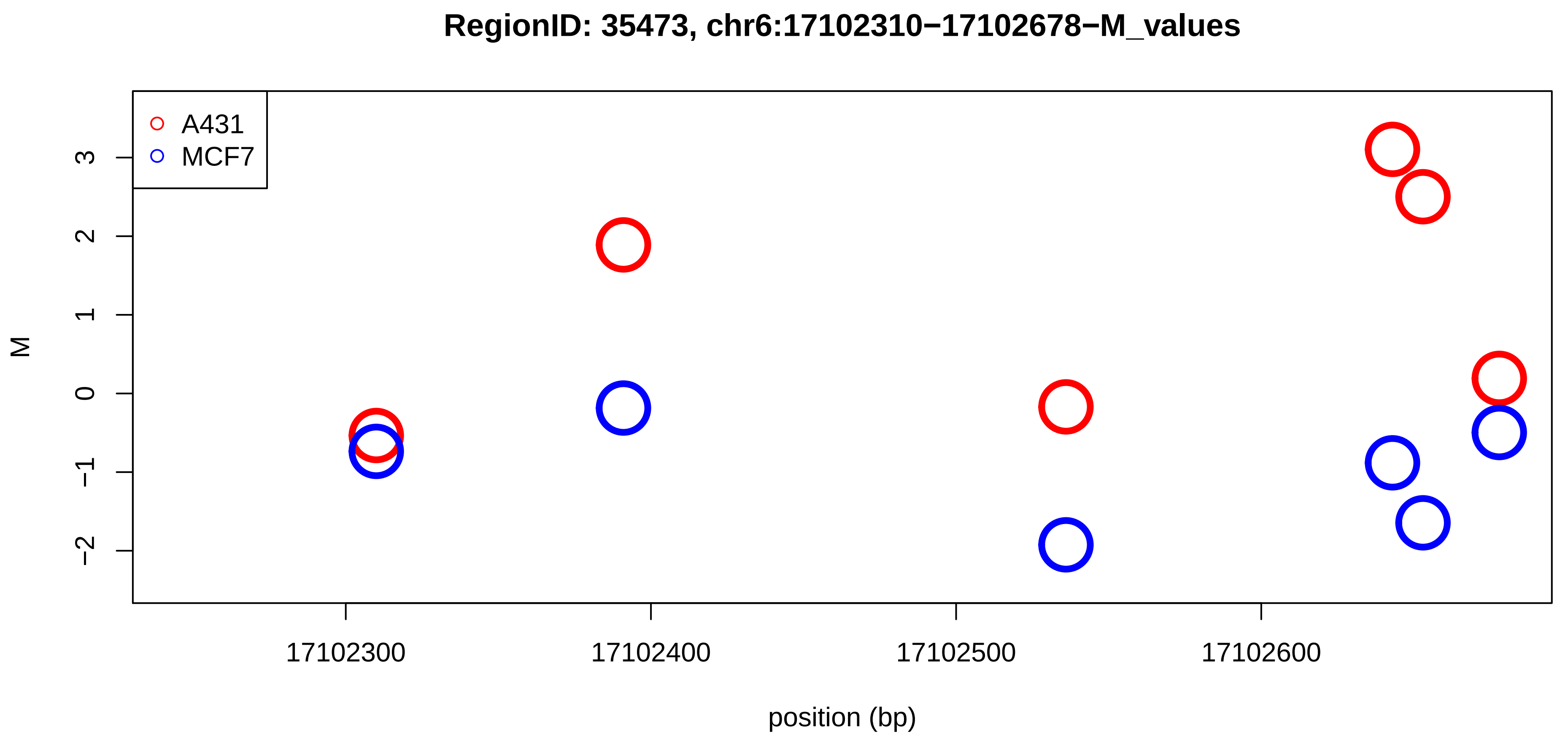
RegionID: 35473, chr6:17102310−17102678−M_values
A431
MCF7
M
3
2
1
0
−1
−2
17102300
17102400
17102500
17102600
position (bp)

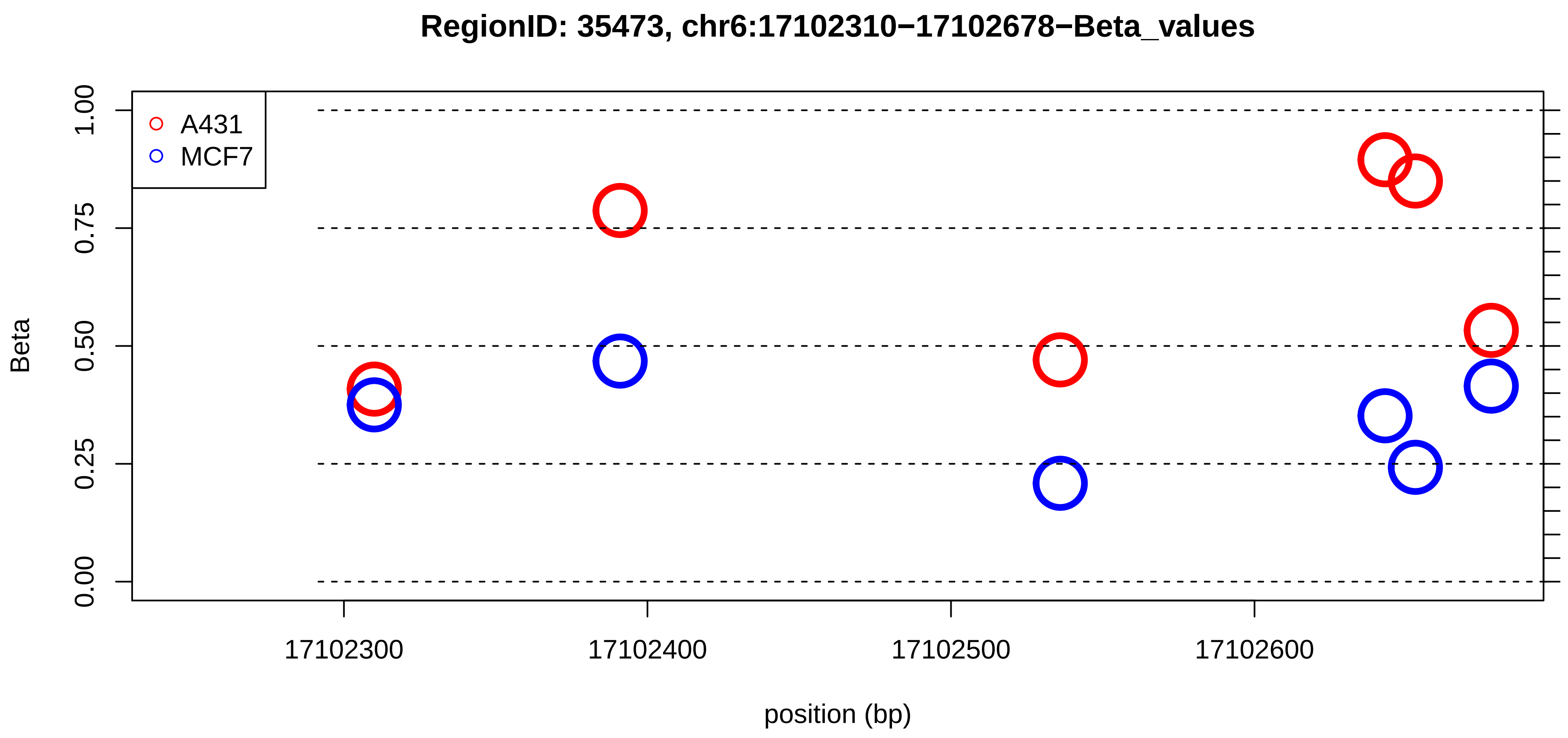
RegionID: 35473, chr6:17102310−17102678−Beta_values
A431
MCF7
Beta
1.00
0.75
0.50
0.25
0.00
17102300
17102400
17102500
17102600
position (bp)

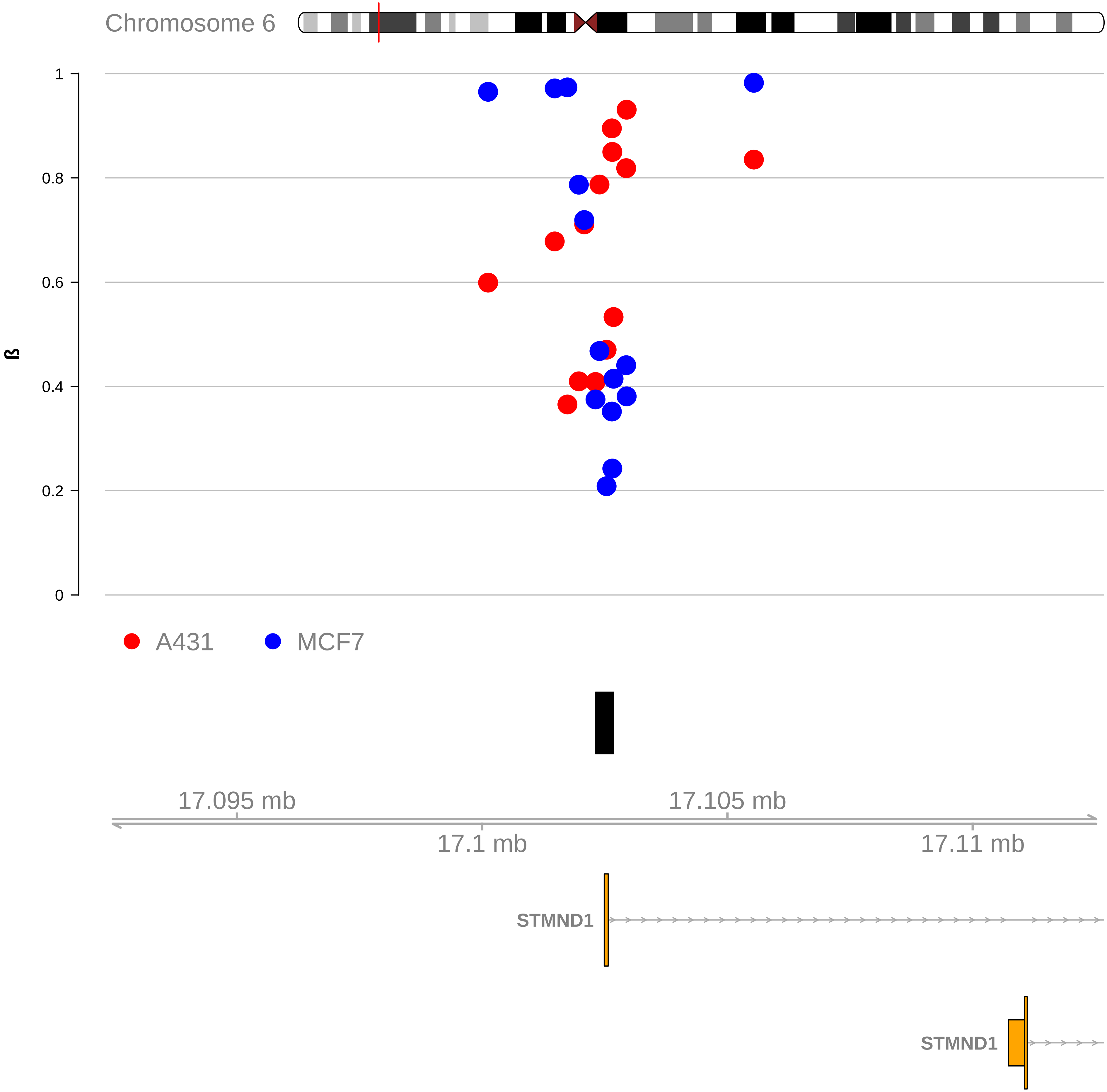

Chromosome 6
1
0.8
0.6
0.4
0.2
0
ß
A431
MCF7
17.095 mb
17.105 mb
17.1 mb
17.11 mb
STMND1
STMND1
